# In-hospital mortality associated with necrotizing soft tissue infection due to *Vibrio vulnificus*: a matched-pair cohort study

**DOI:** 10.1186/s13017-022-00433-z

**Published:** 2022-05-27

**Authors:** Chih-Yao Chang, Kai-Hsiang Wu, Po-Han Wu, Shang-Kai Hung, Cheng-Ting Hsiao, Shu-Ruei Wu, Chia-Peng Chang

**Affiliations:** 1 Department of Emergency Medicine, Chang Gung Memorial Hospital, Chiayi, Taiwan; 2 Department of Emergency Medicine, Chang Gung Memorial Hospital, Taoyüan, Taiwan; 3 Department of Medicine, Chang Gung University, Taoyüan, Taiwan; 4 Department of Pediatric, Kaohsiung Veterans General Hospital, Kaohsiung, Taiwan

**Keywords:** Necrotizing soft tissue infection, Mortality, *Vibrio vulnificus*

## Abstract

**Background:**

It remains unclear whether *Vibrio vulnificus* necrotizing soft tissue infection (NSTI) is associated with higher mortality compared with non-*Vibrio* NSTI. This study’s objective was to compare outcomes including in-hospital mortality and prognosis between patients with *V. vulnificus* NSTI and those with non-*Vibrio* NSTI.

**Method:**

A retrospective 1:2 matched-pair cohort study of hospitalized patients with NSTI diagnosed by surgical finding was conducted in two tertiary hospitals in southern Taiwan between January 2015 and January 2020. In-hospital outcomes (mortality, length of stay) were compared between patients with and without *V. vulnificus* infection. We performed multiple imputation using chained equations followed by multivariable regression analyses fitted with generalized estimating equations to account for clustering within matched pairs. All-cause in-hospital mortality and length of stay during hospitalization were compared for NSTI patients with and without *V. vulnificus*.

**Result:**

A total of 135 patients were included, 45 in *V. vulnificus* NSTI group and 90 in non-*Vibrio* group. The *V. vulnificus* NSTI patients had higher mortality and longer hospital stays. Multivariable logistic regression analysis revealed that *V. vulnificus* NSTI was significantly associated with higher in-hospital mortality compared with non-*Vibrio* NSTI (adjusted odds ratio = 1.52; 95% confidence interval 1.36–1.70; *p* < 0.01).

**Conclusion:**

*Vibrio vulnificus* NSTI was associated with higher in-hospital mortality and longer hospital stay which may increase health care costs, suggesting that preventing *V. vulnificus* infection is essential.

## Introduction

Necrotizing soft tissue infection (NSTI) is a severe, rapidly progressive disease that is characterized by the infection of subcutaneous tissue and fascia, resulting in extensive fascial necrosis [1]. The gold standard management for NSTI is rapid debridement and broad-spectrum antibiotics [2]. Even under rapid and timely management, the risk of mortality and morbidity, such as amputation and multiorgan dysfunction, remains high [3–5]. NSTI caused by *Vibrio vulnificus* is a life-threatening condition with rapid progression and high mortality. It usually occurs through the injuries sustained when handling seafood, wound exposure to seawater, and ingestion of contaminated under-cooked seafood. Immunocompromised patients are also at higher risk of developing this disease [6–8].

Mortality from NSTI caused by gram-negative pathogens has been reported to be higher than caused by other pathogens in warm costal area [9]; however, it remains unclear whether mortality from *V. vulnificus* NSTI is higher than mortality from non-*Vibrio* NSTI. Only a few studies have compared mortality between *V. vulnificus* NSTI and non-*Vibrio* NSTI; one showed that the clinical features of *V. vulnificus* infection were more rapidly progressive and fulminant than those of the methicillin-resistant *Staphylococcus aureus* (MRSA) or MSSA infection [10], whereas another showed that the laboratory risk indicator for necrotizing fasciitis (LRINEC) score was inaccurate in necrotizing fasciitis caused by *V. vulnificus.* [11]. The aim of the present study was to examine the differences in in-hospital mortality between patients with *V. vulnificus* NSTI and those with non-*Vibrio* NSTI, adjusting for comorbidities.

## Material and methods

### Patient selection

Under the approval of institutional review board, a retrospective cohort study was conducted. The medical records of patients who met the inclusion criteria of surgically proven NSTI and who received management between 2015 and 2020 in two tertiary hospitals were reviewed. Among the patients with NSTI, we selected *V. vulnificus* group and non-*Vibrio* group with 1:2 matching: For each patient in the *V. vulnificus* group, we identified two non-*Vibrio* patients of the same sex who were admitted to the same hospital in the same year and whose ages were within 5 years of the age of the *V. vulnificus* patient. We defined *V. vulnificus* NSTI group as blood culture or surgical wound culture yields *V. vulnificus,* or both yield *V. vulnificus.*

### Data collection and analysis

Age, sex, vital signs in the emergency department (ED), hospital stay, the presence of comorbidities, and blood biochemistry profile were analyzed. After data collection was completed, random chart reviews were performed to ensure accuracy. We used hospital identifiers for matching to cancel out site-specific effects such as physician practice patterns and treatment outcomes. We performed multiple imputation for missing data on systolic blood pressure, body temperature, heart rate, and biochemistry analysis of blood. We replaced each missing value with a set of substituted plausible values by generating multiple datasets using the multivariate imputation by chained equations method. The following covariates were used to create these 10 complete datasets: *V. vulnificus* NSTI, age, sex, comorbidity, seawater or seafood contact, serum lactate, LRINEC (laboratory risk indicator for necrotizing fasciitis) sore, in-hospital death, and 30-day in-hospital death, with the assumption that data were missing at random. Estimates from these imputed datasets were combined using Rubin’s rule to obtain combined imputation estimates and standard errors. Then, using multivariable logistic regression analysis fitted with generalized estimating equations to account for the 1:2 matched-pair clustering, we examined the factors associated with all-cause in-hospital mortality.

### Outcomes

The primary outcome of this study was all-cause in-hospital mortality. The secondary outcomes were 30-day in-hospital mortality and length of stay.

### Statistical analysis

All data were analyzed using the Statistical Package for the Social Sciences software, version 20.0 (IBM Corp., Armonk, NY, USA). The chi-square test was used to compare proportions between groups. The two-sample *t* test was used to compare average values, and the Mann–Whitney test was used to compare the median values between groups. Using multivariable logistic regression analysis fitted with generalized estimating equations to account for the 1:2 matched-pair clustering, we examined the factors associated with all-cause in-hospital mortality. Multiple linear regression analysis fitted with generalized estimating equations.

## Results

A total of 135 patients were included, 45 in *V. vulnificus* NSTI group and 90 in non-*Vibrio* group. The mean age was 69.3 years (standard deviation [SD] = 12.7), and 66.4% of the patients were male. Table 1 shows the characteristics of patients with *V. vulnificus* NSTI and non-*Vibrio* NSTI after 1:2 matching. The *V. vulnificus* NSTI group tended to have hypotension, bacteremia, history of seawater or seafood contact, comorbidity with liver disease, and higher serum lactate. All-cause in-hospital mortality was 13.3% in the *V. vulnificus* NSTI, whereas it was 7.8% in the non-*Vibrio* NSTI (Table 2.). All-cause 30-day was also higher in the *V. vulnificus* NSTI than in the non-*Vibrio* group. Length of hospital stay was longer in the *V. vulnificus* NSTI than in the non-*Vibrio* group. Table 3 shows the results of the multivariable logistic regression analysis with generalized estimating equations after multiple imputation for all-cause in-hospital mortality. *V. vulnificus* NSTI was significantly associated with higher mortality compared with non-*Vibrio* group (adjusted odds ratio = 1.52; 95% confidence interval 1.36–1.70; *p* < 0.01). Higher mortality was significantly associated with higher serum lactate, higher LRINEC score, and with liver disease.

**Table 1:** Clinical characteristics between *V. vulnificus* and non-*Vibrio* groups

Variables	*V. vulnificus* group	Non-*Vibrio* group	*P* value
	*N* (%)	*N* (%)	
Age (years, mean ± SD)	69.1 ± 12.8	69.4 ± 12.5	0.82
Sex (male)	29 (64.4%)	58 (64.4%)	0.99
Systolic blood pressure < 90 mmHg	10 (22.2%)	7 (7.7%)	0.02^*^
Heart rate ≧ 100/min	24 (53.3%)	44 (48.9%)	0.59
Body temperature > 38 °C	11 (24.4%)	23 (25.6%)	0.38
Malignancy	3(6.7%)	5 (5.6%)	0.63
Heart disease	7 (15.6%)	12 (13.3%)	0.57
Liver disease	19 (42.2%)	28 (31.1%)	< 0.03^*^
Kidney disease	13 (28.9%)	23 (25.6%)	0.72
Peripheral vascular disease	4 (8.9%)	7 (7.8%)	0.41
Diabetes mellitus	12 (26.7%)	26 (28.9%)	0.28
Presence of bacteremia	29 (64.4%)	19 (21.1%)	< 0.01^*^
Seawater or seafood contact	40 (88.9%)	11 (12.2%)	< 0.01^*^
CRP (mg/L) (mean)	70.1 ± 78.9	98.6 ± 95.1	0.08
WBC (cells/mm^3^) (mean)	14,870 ± 10,960	15,870 ± 9150	0.46
Hemoglobin (g/dL) (mean)	12.5 ± 2.12	12.1 ± 1.68	0.29
Blood glucose (mg/dL) (mean)	168 ± 74	196 ± 108	0.54
Sodium (mmol/L) (mean)	135 ± 2.5	136 ± 2.2	0.18
Lactate (mg/dL) (mean)	16.3 ± 7.8	12.8 ± 10.5	0.03^*^
Creatinine (mg/dL) (mean)	1.58 ± 1.15	1.32 ± 0.76	0.84

*CRP* C-reactive protein, *WBC* white blood cell

*P < 0.05

**Table 2:** Outcomes of patients with *V. vulnificus* and non-*Vibrio* after 1:2 matching

	*V. vulnificus *group	Non-*Vibrio* group	*P* value
In-hospital mortality	6 (13.3%)	7 (7.8%)	< 0.01^*^
30-day mortality	5 (11.1%)	5 (5.6%)	< 0.01^*^
Length of hospital stay (days)	28 (18–46)	20 (14–53)	0.02^*^

Data are expressed as numbers (%) or as medians [interquartile ranges]

*P < 0.05

**Table 3:** Multivariable logistic regression analysis with generalized estimating equations accounting for clustering within matched pairs for all-cause in-hospital mortality

	Adjusted odds ratio	95% confidence interval	*P* value
*V. vulnificus* NSTI	1.52	1.36–1.70	< 0.01^*^
Age (years)	1.01	1.00–1.01	< 0.01^*^
Sex (male)	0.59	0.52–0.67	0.36
Liver disease	1.42	1.08–1.87	< 0.01^*^
Kidney disease	0.95	0.68–1.32	0.74
Seawater or seafood contact	1.40	1.01–1.97	< 0.01^*^
Lactate > 18 mg/dL	1.59	1.07–2.38	< 0.01^*^
Lactate ≦ 18 mg/dL	1.09	0.72–1.63	0.48
LRINEC score > 8	1.84	1.54–2.20	< 0.01^*^
LRINEC score ≦ 8	1.05	0.80–1.37	0.69

*P < 0.05

## Discussion

*Vibrio vulnificus* is a gram-negative marine bacterium that is usually present in warm coastal waters. The main clinical manifestations of *V. vulnificus* infections in humans are gastrointestinal illnesses, primary septicemia, and wound infections. The clinical course can progress rapidly by releasing hemolysins and proteases and result in hemorrhagic bullae and severe skin necrosis [10, 12, 13]. The routes of necrotizing soft tissue infection caused by *V. vulnificus* include wound infections while handling seafood, exposure of a preexisting wound to seawater, and ingestion of contaminated under-cooked seafood [12–14].

Using two tertiary hospital database in Southern Taiwan, our study showed that mortality was higher in patients with *V. vulnificus* NSTI than those in non-*Vibrio* group. In our study, in-hospital mortality among patients with *V. vulnificus* NSTI was 13.3%. Previous studies have reported *vulnificus* NSTI mortality around 10–13% [9, 15], which is comparable to our results. Mortality from NSTI caused by gram-negative or monomicrobial pathogen has been reported to be higher than caused by other pathogens. Namany et al. [16] found that monomicrobial disease group had a significantly higher 90-day mortality rate in addition to higher rates of in-hospital mortality, ICU admission, and vasopressor use than the polymicrobial disease group. Huang et al. [17] revealed that NSTI caused by monomicrobial *Aeromonas* spp. revealed high mortality rates, even through aggressive surgical debridement and antibacterial therapies. They all raised concern for increasing risk of monomicrobial infection and gram-negative pathogen. As comparison, the non-*Vibrio* group are including gram-positive, negative, and culture no growth patients, and possibly monomicrobial or polymicrobial. Mainly pathogens included *Aeromonas* spp., *Pseudomonas*, *Escherichia coli*, *Klebsiella pneumoniae*, *Staphylococcus*, and *Streptococcus* spp. (There are 39 monomicrobial gram-positive, 10 monomicrobial negative, 15 polymicrobial, and 26 culture no growth in non-*Vibrio* group in our data.)

In *V. vulnificus* NSTI, there are 17 patients with positive blood and wound culture (37.8%), 12 patients with positive blood culture (26.7%), and 16 patients with positive wound culture (35.5%). In non-*Vibrio* group, there are 19 patients with bacteremia (21.1%). *V. vulnificus* group had higher incidence of bacteremia than non-*Vibrio* group (64.4% vs 21.1%). Tsai et.al conducted a study about NSTI patients with *V. vulnificus* and methicillin-resistant *Staphylococcus aureus* (MRSA). The incidence of bacteremia was 65% vs 18.75%; *V. vulnificus* group was also higher than MRSA group [9]. Immune-compromised and chronic liver disease (chronic hepatitis, liver cirrhosis, and hepatocellular carcinoma) patients with *V. vulnificus* are more likely to develop blood stream infections [6, 18, 19]. In our study, the incidence of chronic liver disease was also higher in *V. vulnificus* group*. Vibrio vulnificus* can produce *Vibrio vulnificus* hemolysin/cytolysin (VVH), multifunctional autoprocessing RTX toxin, *Vibrio vulnificus* serine protease (VvsA), and *Vibrio vulnificus* protease (VVP). Both VVH and RTX toxin contribute to bacterial invasion to the blood stream and play a role in the development of hypotensive septic shock. The RTX toxin may promote the colonization at the primary infection site and cause severe tissue damage by releasing degrading cytotoxins to induce the inflammatory responses, that *Vibrio vulnificus* can then spread to the blood stream to result in systemic manifestations. Both VVP and VvsA are produced in the interstitial tissues of limbs and cause serious collagenolytic, hemorrhagic, or edematous skin damage in the extremities [9, 20–23]. The specific toxin of *V. vulnificus* may explain a relatively higher rate of blood steam infection than other pathogens. However, the comparison of *Vibrio vulnificus* bacteremia between NSTI and food-borne infection settings needs further studies.

Studies have shown conflicting results on the difference in mortality between patients with NSTI in subgroups. One study had shown higher mortality for NSTI patients with *Aeromonas* species than for those with *V. vulnificus* [24]. One study had shown no significant differences of mortality between NSTI patients with *V. vulnificus* and *Klebsiella pneumoniae* [25]. Another recent study has shown no significant differences of mortality between NSTI patients with *V. vulnificus* and MRSA [9]. But few studies have focused on *V. vulnificus* versus non-*Vibrio* NSTI. In the present study, we clearly demonstrated that mortality was higher in patients with *V. vulnificus* than in patients with non-*Vibrio* NSTI. Besides, longer hospital stay may lead to higher hospitalization cost in *V. vulnificus* NSTI.

Our study showed that patients with hepatic dysfunction had significant associations with *V. vulnificus* infections and mortality. There were studies considered liver cirrhosis as risk factor with increased mortality among NSTI patients [9, 26–29]. Previous study had demonstrated that hyperlactatemia is significantly associated with in-hospital mortality in NSTI patients, even after adjusting for acidosis [30, 31]. The result was similar in the present study, even in *V. vulnificus* infection. Because NSTI and its rapidly progressive infection remain associated with high mortality, the LRINEC score, developed by Wong et al. [32] based on readily available laboratory markers, has been consistently evaluated for its efficacy in various studies. One study has been reported to be inaccurate in necrotizing fasciitis caused by *V. vulnificus* [11]. However, we found the higher LRINEC score may not help early diagnosis with *V. vulnificus* NSTI but display a significant role with prognosis, associated with higher mortality in *V. vulnificus* NSTI patients.

This study still has some limitations. First, this study lacks external validation. The validity of the result still requires other studies to confirm. Second, the retrospective design of this study had its inherent limitation in data collection, such as unverified comorbidities. Third, the sample size is relatively small due to the rarity of NSTI. Fourth, the degree of liver disease in each group is not clearly stated.

## Conclusions

In summary, adjusted in-hospital mortality was significantly higher for patients with *V. vulnificus* NSTI than for those with non-*Vibrio* NSTI in this matched-pair cohort study. Preventing *V. vulnificus* infection is essential. The validity of the result still needs to be confirmed by further studies.

## Data Availability

All the data will be available upon motivated request to the corresponding author of the present paper.

## Abbreviations

CRP: C-reactive protein
ED: Emergency department
Hb: Hemoglobin
LRINEC: Laboratory risk indicator for necrotizing fasciitis
MRSA: Methicillin-resistant *Staphylococcus **aureus*
NSTI: Necrotizing soft tissue infection
OR: Odds ratio
SD: Standard deviation
*V. **vulnificus*: *Vibrio* *vulnificus*
WBC: White blood cell
